# Is occupational noise associated with arthritis? Cross-sectional evidence from US population

**DOI:** 10.1186/s12889-024-17897-0

**Published:** 2024-02-05

**Authors:** Zhounan Wu, Yuhang Liang, Ammna Khan, Jinshen He

**Affiliations:** 1 Department of Orthopaedic Surgery, the Third Xiangya Hospital of Central South University, 138 Tongzipo Road, Hexi Yuelu District, Changsha, Hunan 410013 China; 2 Xiangya School of Medicine, Central South University, Changsha, 410013 China

**Keywords:** NHANES, Arthritis, Occupational noise exposure, Cross-sectional study, Osteoarthritis, Rheumatoid arthritis

## Abstract

**Background:**

The impact of occupational noise exposure on various diseases, including ear and cardiovascular diseases, has been studied extensively. Nevertheless, the connection between osteoarthritis (OA) and rheumatoid arthritis (RA) and occupational noise exposure remains largely unexplored in real-world scenarios. This study assessed the association between occupational noise exposure and the prevalence of two types of arthritis.

**Methods:**

This study used database data from 2005 to 2012 and 2015–March 2020 from the prepandemic National Health and Nutrition Examination Survey (NHANES) related to occupational noise exposure and arthritis. Multivariate logistic regression analysis was used to estimate the association between occupational noise exposure and RA/OA, adjusting for age, gender, race, education level, marital status, the ratio of family income to poverty, trouble sleeping, smoking status, alcohol consumption, diabetes, hypertension, body mass index (BMI), metabolic equivalents (METs), and thyroid disease.

**Results:**

This study included 11,053 participants. Multivariate logistic regression analysis demonstrated that previous exposure to occupational noise was positively associated with self-reported RA (OR = 1.43, 95% CI = 1.18–1.73) and OA (OR = 1.25, 95% CI = 1.07–1.46). Compared to individuals without a history of occupational noise exposure, those with an exposure duration of 1 year or greater exhibited higher odds of prevalent RA, though there was no apparent exposure response relationship for noise exposure durations longer than 1 year. The results of our subgroup analyses showed a significant interaction between age and occupational noise exposure on the odds of self-reported prevalent OA.

**Conclusions:**

Our findings suggest an association between occupational noise exposure and the prevalence of RA and OA. Nevertheless, further clinical and basic research is warranted to better explore their associations.

**Supplementary Information:**

The online version contains supplementary material available at 10.1186/s12889-024-17897-0.

## Introduction

Osteoarthritis (OA) and rheumatoid arthritis (RA) are prevailing inflammatory joint disorders that have a considerable impact on the population and are mainly characterized by joint dysfunction, ache, and restricted physical activity [1, 2]; extensive research has revealed numerous risk factors associated with the development of these conditions [3, 4]. Currently, the role of occupational exposure (radiation, nanomaterials, lead exposure, etc.) in the risk of OA and RA is widely recognized [5, 6], yet insufficient attention has been given to occupational noise, which is one of the most common exposures at the workplace [7]. Many studies have firmly established the link between occupational noise and the onset of detrimental auditory health effects [7–9], as well as nonauditory health implications [6, 10–12]. However, there is still uncertainty about whether OA and RA are associated with occupational noise exposure.

Although we proposed an underlying mechanistic hypothesis in this study, there is little evidence to directly suggest risks associated with occupational noise exposure and arthritis, and there is no consensus on this issue. A study among Brazilian individuals using the National Survey on Health showed that exposure to noise in the workplace contributed 2.16 times to the threat of musculoskeletal disorders (95% CI: 1.68–2.77) [13]. The scientific validity of the above study was further supported by the findings of an animal study indicating that prolonged noise stress increases both the incidence and early severity of collagen-related arthritis in rats [14]. A previous investigation based on the 2011–2012 cycle sample of the National Health and Nutrition Examination Survey (NHANES) revealed a significant association between prolonged exposure to intense noise and self-reported RA [15]. In contrast, certain studies demonstrated no significant increase in the prevalence of arthritis among respondents exposed to noise [16]. Moreover, the previous studies used data that were somewhat outdated or not conducted in the United States, so whether their conclusions can be generalized to Americans is debatable.

Several studies have suggested that noise might, through the neural pathway, induce oxidative stress and the production of inflammatory factors [17, 18], potentially leading to inflammation [19, 20], which could be a common mechanism for the development of OA and RA. Since both types of arthritis mentioned above are common in noise-exposed occupations and because their underlying pathogenic mechanisms are related, we wanted to study these two diseases together and analyse the impact of occupational noise exposure on them. Therefore, we assessed the association between occupational noise exposure and two subtypes of arthritis, OA and RA, in this study using a comprehensive fraction of individuals from the NHANES. It was hypothesized that occupational noise exposure is significantly associated with an increase in the prevalence of both RA and OA within the general US population.

## Materials and methods

### Study design

The NHANES was initiated in the early 1960s and has been continuously conducted. The NHANES is a yearly examination of a sample of approximately 10,000 individuals, representing the noninstitutionalized population of the United States and excluding active military personnel, incarcerated individuals, and people in group homes. The data collected through NHANES interviews provide comprehensive information on various aspects, such as socioeconomic status, demographic characteristics, dietary habits, and health issues. The wealth of data provided by the NHANES has led to the publication of numerous research articles [21–23], contributing to a better comprehension of various health issues and their determinants in the population.

### Study population

**Fig. 1:**
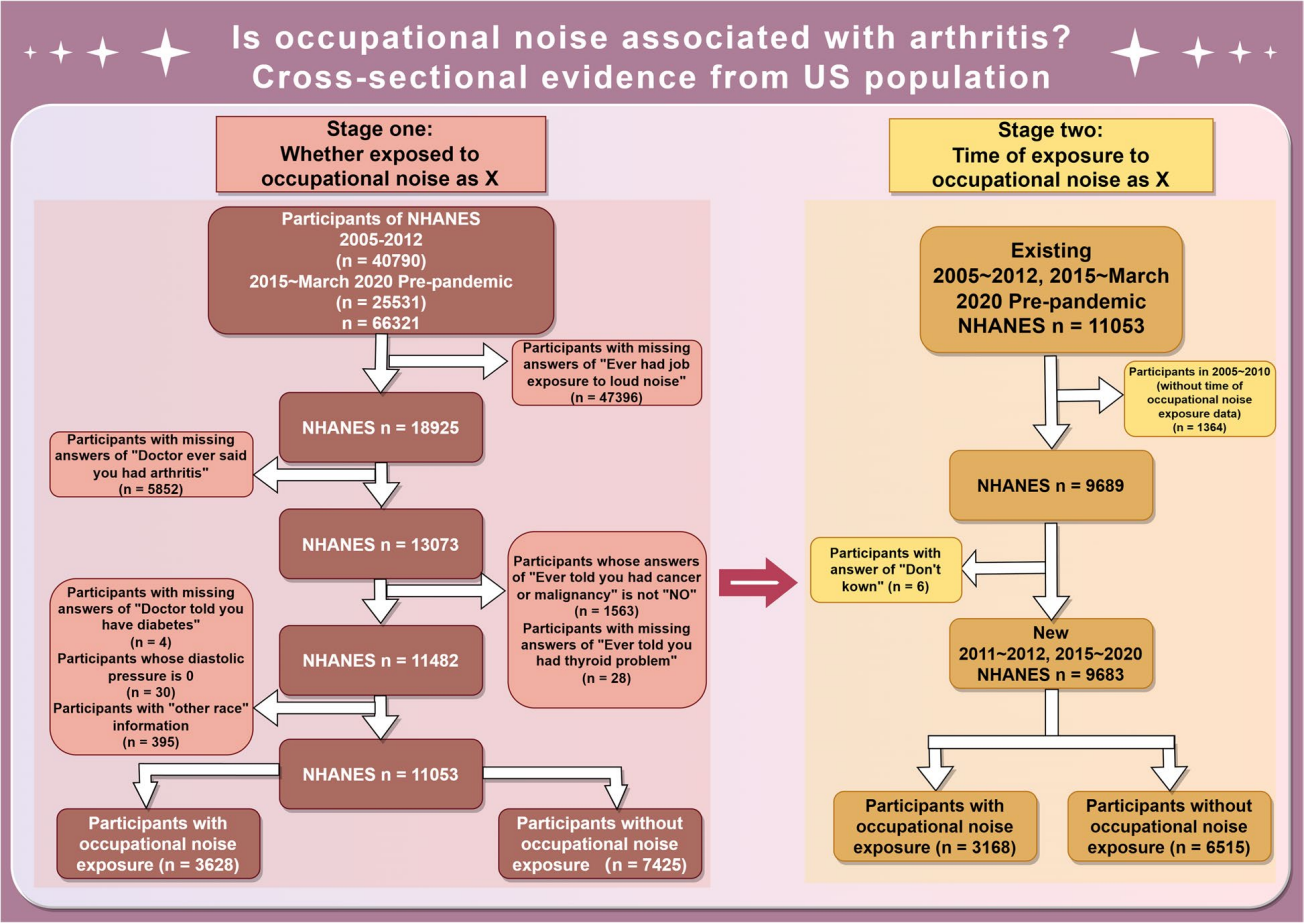
Flow chart of sample selection from the NHANES 2005–2012, 2015-March 2020 Pre-pandemic

Two cohorts were included in this study; the first cohort was constructed to explore the association between presence or absence of occupational noise exposure and RA/OA, while second cohort was constructed to examine whether duration of noise exposure in years impacted observed associations. The first cohort consisted of individuals sourced from the prepandemic 2005–2012 and 2015-March 2020 NHANES cycles (left panel of Fig. 1). Participants with missing occupational noise exposure information (*n* = 47,396), missing arthritis information (*n* = 5852), missing thyroid problem information (*n* = 28), missing diabetes information (*n* = 4), a history of cancer or malignancy, as well as those with missing answers regarding this issue (*n* = 1563), participants whose diastolic blood pressure was 0 (*n* = 30), or participants categorized as “other races” (*n* = 395), were excluded from this study. Following the necessary exclusions, the final analysis included a total of 11,053 participants. Specifically, tumour patients were excluded because cohort studies have shown a strong association between cancer and inflammatory arthritis [24]. In addition, data related to occupational noise exposure from 2013 to 2014 were not published in the NHANES; therefore, this research used data from 2005 to 2012 and 2015 onwards rather than continuous data.

Given that occupational noise exposure duration data were only reported by the NHANES after 2011, the second cohort was a sub-set of the first cohort, focusing on respondents from 2011 to 2012 and 2015-March 2020 to explore the association between occupational noise exposure duration and arthritis. We excluded participants from 2005 to 2009 as well as six respondents with missing exposure duration data, resulting in a total of 9,683 participants (right panel of Fig. 1). Written consent was received from all respondents prior to questionnaire collection. The NHANES protocols were reviewed and approved by the ethics review boards of the NCHS and CDC (https://www.cdc.gov/nchs/nhanes/irba98.htm).

### Variables

The exposure variables in this study included ever having had occupational exposure to loud noise and the duration of exposure to loud noise. Exposure to loud noise at work was defined as having ever had a job or a combination of jobs that exposed someone to loud noise for four or more hours a day, several days a week. Loud noise meant that a participant needed to raise the volume of their voice to be heard by others. Participants who reported ‘No’ or ‘Never worked’ were categorized as controls, and those who reported ‘Yes’ were categorized as having occupational noise exposure. The duration of occupational noise exposure referred to the number of months or years participants had been exposed to high-volume or noisy conditions at work for at least four hours per day, several days per week. Based on the duration of occupational noise exposure, we classified individuals into five categories: no exposure to occupational noise, less than 1 year, 1–4 years, 5–14 years, and 15 years or more. Computer-assisted personal interviews (CAPIs) were used to obtain detailed self-reported data.

The outcome variable was the presence of OA or RA. The questions for outcome variables included “Has a doctor ever said you had arthritis?” and “Which type of arthritis was it?”. Participants who answered “yes” to the first question were asked to answer a question about the type of arthritis, and the responses might include “Osteoarthritis or degenerative arthritis”, “Rheumatoid arthritis”, “Psoriatic arthritis”, “other” or “Refuse to answer/Do not know”. CAPIs were also used for quality control. More detailed information can be found at https://wwwn.cdc.gov/Nchs/Nhanes/2015-2016/MCQ_I.htm.

**Fig. 2:**
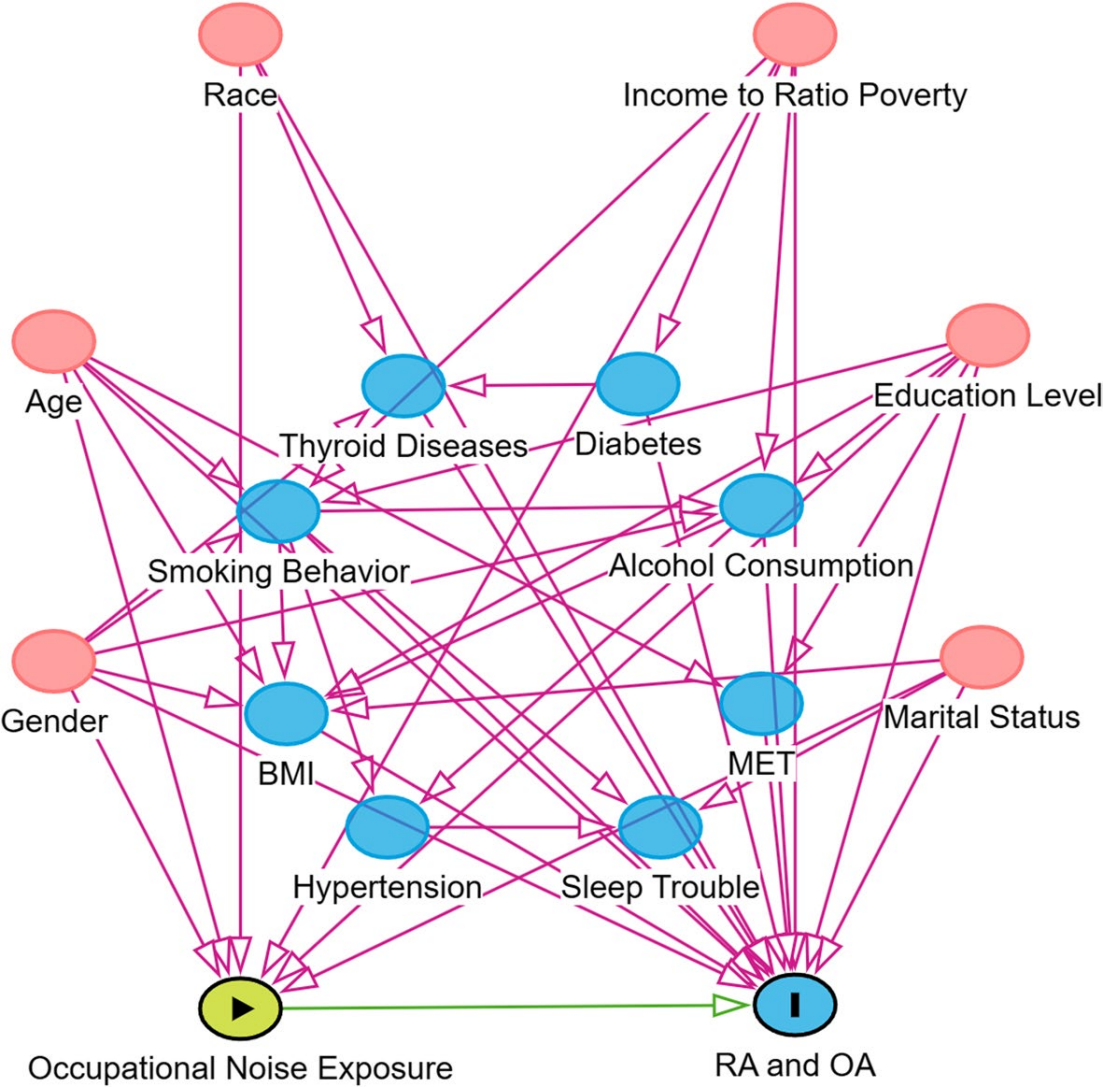
Directed Acyclic Graph (DAG). DAG plot shows the effects of confounding on the primary exposures and outcomes. Red circles represent ancestors of the exposure and outcome (i.e., confounders); Blue circles represent ancestors of the outcome (i.e., causal determinants of the outcome); Circle with a triangle represent primary exposure; Circle with a “I” represent primary outcome; Green lines represent causal paths, and pink lines represent biasing paths. The final minimally sufficient adjustment set comprised Gender, Age, Race, The ratio of family income to poverty, Education Level and Marital Status. MET, metabolic equivalent; BMI, body mass index; RA, Rheumatoid arthritis; OA, Osteoarthritis

Potential confounders and effect modifiers were identified from the literature [4, 25–27], and directed acyclic graphs (DAGs) were drawn using the R package DAGitty to guide model construction [28] (Fig. 2). The categorical variables included gender, race, marital status, education level, alcohol consumption, cigarette smoking status, trouble sleeping, hypertension, thyroid problems and diabetes. The continuous variables included age, the ratio of family income to poverty, metabolic equivalents (METs) and body mass index (BMI). Respondents were categorized into four distinct groups based on their ethnicity (Latino, Non-Hispanic white, African American, Asian), with “Latino” including those of Mexican heritage and other Hispanic individuals [29]. Marital status was grouped into “married or living with partner” and “unmarried” (widowed, divorced, separated, or never married). There were two answer options for education: “less than high school” included those with a below grade 12 education and grade 12 without a diploma, while “high school or above” included those who had graduated from high school or above [30]. Hypertension, as measured in the NHANES, was defined as the average blood pressure derived from three or four measurements, with values equal to or exceeding 140 mm Hg for systolic blood pressure or 90 mm Hg for diastolic blood pressure [31]. Participants’ smoking status was assessed by calculating their cumulative consumption of 100 or more cigarettes. Similarly, those who reported consuming 12 or more alcoholic beverages annually were classified as consuming alcohol. To facilitate subgroup analysis, we categorized continuous variables based on insights from prior studies. Age was divided into three brackets: younger than 40 years, between 40 and 60 years, and older than 60 years [32, 33]. BMI was classified as normal (< 25.0 kg/m^2^), overweight (25.0-29.9 kg/m^2^), or obese (≥ 30.0 kg/m^2^) [34, 35]. Physical activity levels were evaluated by calculating questionnaire-based MET scores and grouped into low (< 600 MET-minutes/week), moderate (600–2999 MET-minutes/week), and high (≥ 3000 MET-minutes/week) levels [36]. Further guidance regarding the variables can be accessed on the NHANES website.

### Statistical analysis

To clarify the possible association between occupational noise exposure and two subtypes of arthritis, OA and RA, this study employed various statistical methods. The NHANES database employs a complex probability sampling design, which necessitates weighting the sample to minimize selection bias among age, gender, and race subgroups. Although we screened the data, the distribution of the number of people corresponding to the variables was altered. Therefore, in reference to previous studies [37–39], unweighted estimates were used in this study to explore the relationship between occupational noise exposure and arthritis by incorporating the variables used to calculate sample weights into the adjusted model instead of using weights. Continuous variables are presented as the means and standard deviations, and categorical variables are presented as percentages. The chi-square test was used for categorical variables, and the two-sample t-test was used for continuous variables to compare differences in baseline characteristics between the control and occupational noise-exposed groups. We performed the variance inflation factor (VIF) test on covariables to check for multicollinearity, with a VIF value less than 5 indicating no collinearity [40]. Additionally, a multivariate logistic regression model was used to examine the association between occupational noise exposure and OA/RA, considering potential confounding factors. The unadjusted model (Model 1) did not adjust for any covariates; the minimally adjusted model (Model 2) adjusted for gender, age, race, education level, marital status, and the ratio of family income to poverty; and the fully adjusted model (Model 3) adjusted for age, gender, race, education level, marital status, the ratio of family income to poverty, trouble sleeping, smoking status, alcohol consumption, BMI, diabetes, METs, thyroid disease, and hypertension. The model results were evaluated using odds ratios (ORs) and 95% confidence intervals (CIs). To investigate the relationship between occupational noise exposure and arthritis in different subgroups, subgroup analysis was carried out. Stratification factors included gender (male/female), age (< 40/<60, ≥ 40/≥60 years), race (Latino/Non-Hispanic white/African American/Asian), METs (low/moderate/high) and BMI (normal/overweight/obese). Interaction analysis was used to evaluate the heterogeneity of the associations between the subgroups.

All the abovementioned statistical analyses were conducted using EmpowerStats software (version 2.0) and the R project (version 3.2.3) for statistical calculations. *P* < 0.05 indicated statistical significance.

## Results

The two cohorts included in this study explored the relationship between the presence or absence of occupational noise exposure and the duration of occupational noise exposure and arthritis; these included 11,053 and 9,683 participants aged ≥ 20 years, respectively. The baseline characteristics of the participants according to occupational noise exposure, as a column-stratified variable, are shown in Table 1 and Supplementary Table 1. The VIFs for all covariates ranged from 1.044 to 1.201, which are less than 5; therefore, we detected no covariate collinearity (as shown in Supplementary Table 2). Compared with those in the control group, the respondents exposed to occupational noise tended to be older; male; Non-Hispanic white; married or living with a partner; have a lower education level; low income; have a high BMI; have trouble sleeping; less likely to report thyroid problems; high METs; have diabetes; smoke and consume alcohol; and have RA. In the first cohort, the prevalence of self-reported RA and OA among participants exposed to occupational noise was 6.62% and 10.80%, respectively, whereas it was 4.38% and 9.93%, respectively, in the control group. The details of the prevalence across different age groups are shown in Supplementary Table 3. The statistical results of the prevalence of OA and RA and the number of individuals among the respondents with different occupational noise exposure durations are shown in Supplementary Table 4.

**Table 1:** The characteristics of participants from the first cohort

	Control Group (*n* = 7425)	Occupational Noise Exposure (*n* = 3628)	* P* Value
Osteoarthrosis			0.152
Yes	9.93%	10.80%	
No	90.07%	89.20%	
Rheumatoid arthritis			< 0.001^*^
Yes	4.38%	6.62%	
No	95.62%	93.38%	
Age (year)	50.21 ± 18.87	51.98 ± 18.43	< 0.001^*^
Gender			< 0.001^*^
Male	37.87%	70.31%	
Female	62.13%	29.69%	
Race			< 0.001^*^
Latino	24.79%	26.85%	
Non-Hispanic white	34.81%	43.33%	
African American	23.96%	24.64%	
Asian	16.43%	5.18%	
Education level			< 0.001^*^
Less than high school	20.26%	27.45%	
High school or above	79.74%	72.55%	
Marital status			0.004^*^
Married/Living with Partner	56.79%	59.70%	
Unmarried	43.21%	40.30%	
Body mass index (kg/m^2^)	28.80 ± 6.76	29.60 ± 6.40	< 0.001^*^
Ratio of family income to poverty	2.53 ± 1.56	2.21 ± 1.42	< 0.001^*^
Hypertension			0.013^*^
Yes	12.36%	13.18%	
No	40.79%	42.83%	
Missing	46.95%	43.99%	
Diabetes			< 0.001^*^
Yes	12.28%	16.43%	
No	87.72%	83.57%	
Thyroid problem			< 0.001^*^
Yes	11.15%	8.88%	
No	88.85%	91.12%	
Sleep trouble			< 0.001^*^
Yes	22.87%	26.93%	
No	77.13%	73.07%	
Smoke			< 0.001^*^
Yes	35.42%	54.80%	
No	64.58%	45.20%	
Alcohol			< 0.001^*^
Yes	51.70%	65.46%	
No	29.51%	21.28%	
Missing	18.79%	13.26%	
METs	2542.60 ± 4810.05	5133.10 ± 7807.57	< 0.001^*^

Mean ± SD for continuous variables: *P* value was calculated by the t-test; % for categorical variables, *P* value was calculated by the chi-square test

*MET* Metabolic equivalent

^*^Significant at the *P* < 0.05 level

The results of the multivariate logistic regression analysis between occupational noise exposure and arthritis are displayed in Table 2. Participants who were exposed to occupational noise were more likely to have self-reported RA in the unadjusted model (OR = 1.55, 95% CI = 1.30–1.84), the minimally adjusted model (OR = 1.59, 95% CI: 1.32–1.93), and the fully adjusted model (OR = 1.43, 95% CI: 1.18–1.73). The unadjusted model showed there was no association between occupational noise exposure and self-reported OA, yet the minimally adjusted model (OR = 1.41, 95% CI: 1.22–1.64) and the fully adjusted model (OR = 1.25, 95% CI: 1.07–1.46) did show an association. To better identify the differences between the results of the fully adjusted model overall and the results of the stratified models, the ORs with confidence intervals for the covariates in the adjusted models presented are shown in Supplementary Table 5. As shown in Table 3, this study did not find an association between OA and duration of occupational noise exposure, whereas RA was revealed to be positively associated with duration of occupational noise exposure. Participants with noise exposure durations of 1 year or longer had elevated odds of reporting prevalent RA [1 to 4 years: OR = 1.64, 95% CI = 1.17–2.28; 5 to 14 years: OR = 1.42, 95% CI = 1.02–1.97; 15 years or more: OR = 1.56, 95% CI = 1.15–2.10] compared to those with no history of occupational noise exposure. Since the noise exposure duration categories of 1 year and greater had overlapping confidence intervals, we did not observe dose effects on the association among the participants with exposure durations longer than 1 years.

**Table 2:** Association between occupational noise exposure and rheumatoid arthritis and osteoarthritis

	Model 1	Model 2	Model 3
***Control*** (reference)	1.0	1.0	1.0
***Occupational noise exposure***			
**Self-Reported RA**			
OR (95% CI)	1.55 (1.30, 1.84)	1.59 (1.32, 1.93)	1.43 (1.18, 1.73)
* P* value	< 0.001^*^	< 0.001^*^	< 0.001^*^
**Self-Reported OA**			
OR (95% CI)	1.10 (0.97, 1.25)	1.41 (1.22, 1.64)	1.25 (1.07, 1.46)
*P* value	0.152	< 0.001^*^	0.004^*^

The data: OR (95% CI) *P* value. Unadjusted model (Model 1): no covariates were adjusted; Minimally adjusted model (Model 2): gender, age, race, education level, marital status, ratio of family income to poverty were adjusted; Fully adjusted model (Model 3): age, BMI, alcohol, gender, race, education level, marital status, hypertension, diabetes, ratio of family income to poverty, sleep trouble, smoke, METs, thyroid disease were adjusted

*OR* Odds ratio, *MET* Metabolic equivalent, *BMI* Body mass index, *RA* Rheumatoid arthritis, *OA* Osteoarthritis

^*^Significant at the *P* < 0.05 level

**Table 3:** Association between duration of occupational noise exposure and rheumatoid arthritis and osteoarthritis

	Model 1		Model 2		Model 3	
***Variables***	OR (95%CI)	*P* value	OR (95%CI)	*P* value	OR (95%CI)	*P* value
**Rheumatoid arthritis**						
*** Control*** (reference)	1.0		1.0		1.0	
*** Duration of noise exposure***						
Less than 1 year	0.77 (0.46, 1.28)	0.313	1.04 (0.61, 1.76)	0.890	0.95 (0.56, 1.62)	0.858
1 to 4 years	1.57 (1.15, 2.15)	0.004^*^	1.82 (1.31, 2.52)	< 0.001^*^	1.64 (1.17, 2.28)	0.003^*^
5 to 14 years	1.62 (1.20, 2.20)	0.002^*^	1.62 (1.18, 2.24)	0.003^*^	1.42 (1.02, 1.97)	0.036^*^
15 or more years	2.49 (1.90, 3.27)	< 0.001^*^	1.72 (1.28, 2.32)	< 0.001^*^	1.56 (1.15, 2.10)	0.004^*^
**Osteoarthritis**						
*** Control*** (reference)	1.0		1.0		1.0	
*** Duration of noise exposure***						
Less than 1 year	0.78 (0.55, 1.09)	0.147	1.36 (0.94, 1.96)	0.101	1.29 (0.89, 1.87)	0.183
1 to 4 years	1.02 (0.79, 1.30)	0.900	1.47 (1.12, 1.92)	0.006^*^	1.29 (0.97, 1.70)	0.078
5 to 14 years	1.12 (0.88, 1.42)	0.351	1.34 (1.03, 1.74)	0.028^*^	1.13 (0.86, 1.48)	0.371
15 or more years	1.68 (1.35, 2.08)	< 0.001^*^	1.30 (1.02, 1.65)	0.036^*^	1.17 (0.91, 1.50)	0.215

The data: OR (95% CI) *P* value. Age, BMI, alcohol, gender, race, education level, marital status, hypertension, diabetes, ratio of family income to poverty, sleep trouble, smoke, METs, thyroid disease were adjusted

*OR* Odds ratio, *MET* Metabolic equivalent, *BMI* Body mass index, *RA* Rheumatoid arthritis, *OA* Osteoarthritis

^*^Significant at the *P* < 0.05 level

Subgroup analysis was performed to evaluate the robustness of the association between occupational noise exposure and arthritis. We also tested the interactions with gender, age, race, BMI, and METs. However, the interaction tests revealed no statistically significant differences between strata for the association between occupational noise exposure and RA, suggesting that age, gender, race, BMI, and METs did not significantly influence this positive association (*P* for interaction ≥ 0.05). Our findings indicated a consistent positive association between occupational noise exposure and RA across diverse demographic factors, including gender, age, race, BMI, and METs, suggesting its applicability in various population settings. However, age emerged as a potential factor affecting this association in the stratified analysis between occupational noise exposure and OA (*P* for interaction < 0.05). An association between OA and occupational noise exposure existed for individuals aged between 40 and 60 years (OR = 1.35, 95% CI = 1.01–1.80) and those under the age of 40 (OR = 2.36, 95% CI = 1.32–4.22). The detailed results of the above subgroup analysis stratified by age, race, gender, BMI, and METs are shown in Table 4.

**Table 4:** Subgroup analyses for the association between occupational noise exposure and rheumatoid arthritis/osteoarthritis

		Model 1		Model 2		Model 3		* P* for interaction
***Variables***	**N**	OR (95%CI)	*P* value	OR (95%CI)	*P* value	OR (95%CI)	*P* value	
**Rheumatoid arthritis**								
*** Gender***								0.985
Male	5363	1.97 (1.51, 2.58)	< 0.001*	1.60 (1.22, 2.12)	< 0.001^*^	1.40 (1.05, 1.85)	0.021^*^	
Female	5690	1.62 (1.26, 2.09)	< 0.001*	1.56 (1.20, 2.03)	0.001^*^	1.42 (1.09, 1.86)	0.010^*^	
*** Age***								0.896
< 40	3685	1.61 (0.81, 3.19)	0.176	1.74 (0.84, 3.63)	0.005^*^	1.35 (0.63, 2.88)	0.437	
≥ 40, < 60	3327	1.59 (1.15, 2.19)	0.005^*^	1.66 (1.17, 2.36)	0.005^*^	1.53 (1.07, 2.20)	0.021^*^	
≥ 60	4041	1.42 (1.14, 1.76)	0.002^*^	1.48 (1.17, 1.88)	0.001^*^	1.38 (1.08, 1.75)	0.009^*^	
*** Race***								0.619
Latino	2815	1.24 (0.89, 1.72)	0.208	1.59 (1.11, 2.28)	0.012^*^	1.48 (1.02, 2.14)	0.039^*^	
Non-Hispanic White	4157	1.55 (1.17, 2.06)	0.003^*^	1.61 (1.17, 2.22)	0.004^*^	1.43 (1.03, 1.99)	0.031^*^	
African American	2673	1.43 (1.05, 1.94)	0.022^*^	1.44 (1.03, 2.02)	0.033^*^	1.33 (0.94, 1.88)	0.108	
Asian	1408	2.58 (1.06, 6.27)	0.036^*^	2.00 (0.78, 5.13)	0.151	2.13 (0.78, 5.81)	0.138	
*** BMI***								0.687
< 25.0	3079	1.64 (1.11, 2.42)	0.013^*^	1.35 (0.87, 2.10)	0.181	1.27 (0.81, 1.99)	0.303	
25.0-29.9	4092	1.59 (1.21, 2.09)	0.001^*^	1.75 (1.28, 2.37)	< 0.001^*^	1.63 (1.19, 2.23)	0.002^*^	
≥ 30.0	3882	1.37 (1.05, 1.79)	0.021^*^	1.52 (1.13, 2.03)	0.005^*^	1.35 (1.002, 1.820)^a^	0.049^*^	
*** METs***								0.666
< 600	4578	1.56 (1.23, 1.99)	< 0.001^*^	1.61 (1.24, 2.10)	< 0.001^*^	1.48 (1.13, 1.94)	0.004^*^	
600–2999	3244	2.19 (1.54, 3.13)	< 0.001^*^	1.62 (1.09, 2.41)	0.017^*^	1.48 (0.98, 2.22)	0.060	
≥ 3000	3231	1.35 (0.95, 1.90)	0.090	1.51 (1.03, 2.22)	0.035*	1.40 (0.95, 2.06)	0.092	
**Osteoarthritis**								
*** Gender***								0.104
Male	5363	1.88 (1.51, 2.34)	< 0.001*	1.62 (1.29, 2.04)	< 0.001^*^	1.48 (1.17, 1.87)	0.001^*^	
Female	5690	1.20 (0.99, 1.45)	0.057	1.29 (1.06, 1.59)	0.013^*^	1.12 (0.91, 1.38)	0.287	
*** Age***								< 0.001^*^
< 40	3685	3.23 (1.94, 5.38)	< 0.001*	2.98 (1.71, 5.19)	< 0.001^*^	2.36 (1.32, 4.22)	0.004^*^	
≥ 40, < 60	3327	1.36 (1.06, 1.75)	0.016*	1.47 (1.11, 1.93)	0.007^*^	1.35 (1.01, 1.80)	0.044^*^	
≥ 60	4041	0.82 (0.69, 0.96)	0.017*	1.23 (1.02, 1.49)	0.028^*^	1.14 (0.94, 1.38)	0.190	
*** Race***								0.835
Latino	2815	1.13 (0.84, 1.54)	0.413	1.39 (0.99, 1.95)	0.057	1.22 (0.86, 1.74)	0.268	
Non-Hispanic White	4157	0.88 (0.74, 1.04)	0.139	1.39 (1.14, 1.71)	0.002^*^	1.25 (1.01, 1.55)	0.037^*^	
African American	2673	1.08 (0.81, 1.43)	0.618	1.36 (0.99, 1.86)	0.054	1.21 (0.88, 1.67)	0.248	
Asian	1408	1.59 (0.81, 3.12)	0.181	1.44 (0.69, 2.98)	0.328	1.12 (0.51, 2.42)	0.780	
*** BMI***								0.968
< 25.0	3079	1.08 (0.79, 1.47)	0.638	1.28 (0.89, 1.84)	0.181	1.17 (0.80, 1.71)	0.414	
25.0-29.9	4092	1.03 (0.83, 1.27)	0.800	1.39 (1.09, 1.78)	0.009^*^	1.29 (1.004, 1.660)^a^	0.046^*^	
≥ 30.0	3882	1.07 (0.88, 1.30)	0.508	1.39 (1.12, 1.74)	0.003^*^	1.27 (1.02, 1.60)	0.036^*^	
*** METs***								0.671
< 600	4578	1.13 (0.94, 1.37)	0.194	1.42 (1.15, 1.76)	0.001^*^	1.24 (0.99, 1.54)	0.056	
600–2999	3244	1.37 (1.05, 1.78)	0.019^*^	1.42 (1.05, 1.92)	0.023^*^	1.29 (0.94, 1.76)	0.110	
≥ 3000	3231	1.05 (0.81, 1.36)	0.698	1.34 (1.00, 1.80)	0.051	1.19 (0.88, 1.61)	0.247	

The data: OR (95% CI) *P* value. Model 1: no variables are adjusted; Model 2: age, race, gender, education level, ratio of family income to poverty, marital status are adjusted; Model 3: age, BMI, alcohol, race, education level, marital status, hypertension, diabetes, ratio of family income to poverty, sleep trouble, smoke,

*METs* Thyroid disease were adjusted, *OR* Odds ratio, *MET* Metabolic equivalent, *BMI* Body mass index, *RA* Rheumatoid arthritis, *OA* Osteoarthritis

^*^Significant at the *P* < 0.05 level

^a^Confidence intervals were used with three decimal points to avoid ambiguity

## Discussion

Our study assessed the association between occupational noise exposure and arthritis among adults in the United States utilizing nationally representative data. The odds of self-reported RA was 43% higher among those with a history of occupational noise exposure compared to those with no history of occupational noise exposure, while the odds of self-reported OA was 25% higher. Furthermore, participants with noise exposure durations of 1 year or longer had elevated odds of reporting prevalent RA compared to those with no history of occupational noise exposure. However, no such association was observed for OA. After we adjusted for various covariates and conducted subgroup analyses, we identified age as a potential factor influencing the positive association between occupational noise exposure and OA.

**Fig. 3:**
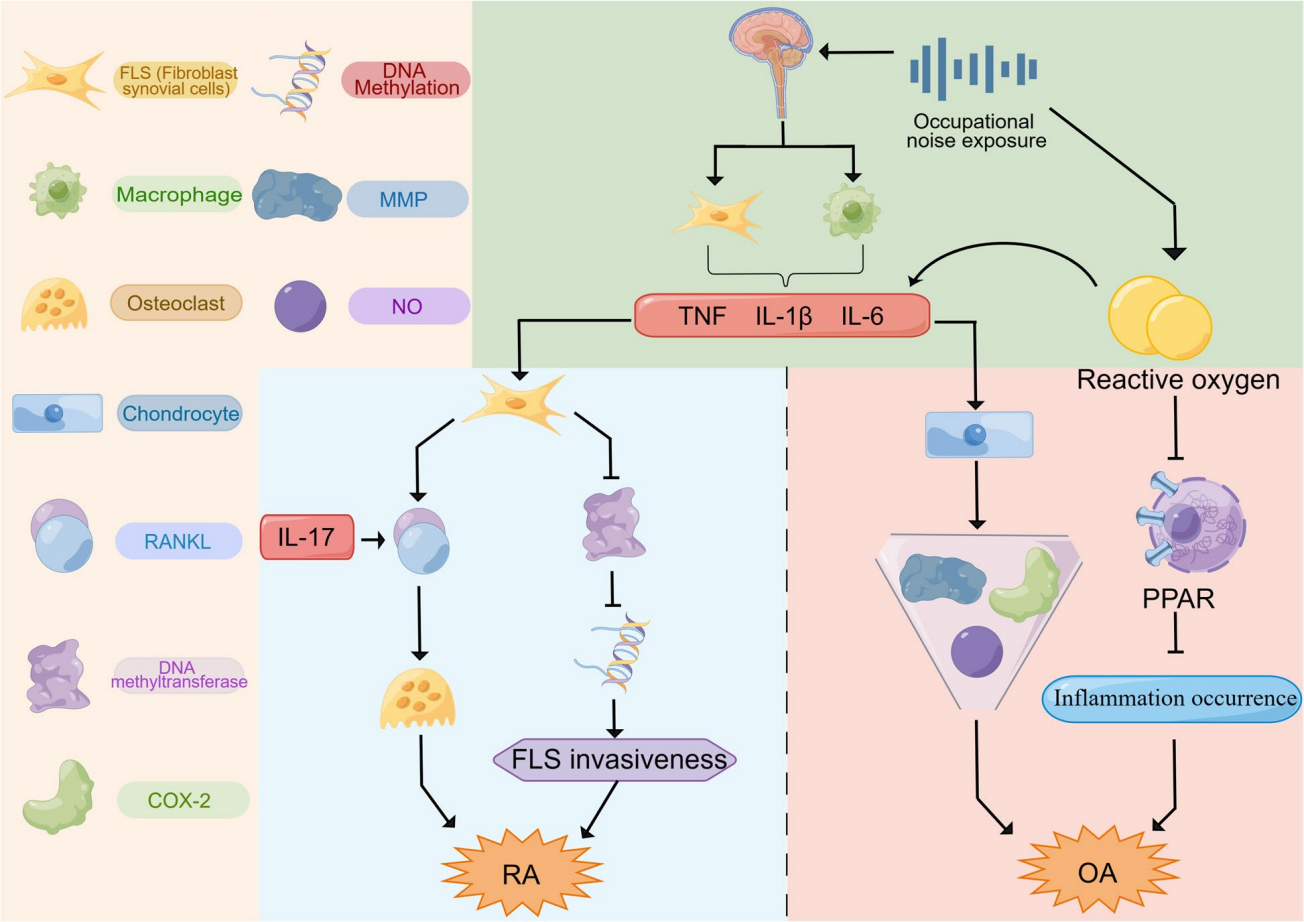
Potential mechanisms of occupational noise exposure leading to RA and OA. Exposure to noise has been linked to the activation of the SNS and the suppression of the PNS, leading to an imbalance in the ANS. This imbalance can influence the activity of α7nAChR receptors found in macrophages and FLS. Normally, activation of these receptors by acetylcholine reduces cytokine production. However, in the case of SNS-PNS imbalance, there may be a reduction in acetylcholine release, potentially resulting in increased production of pro-inflammatory cytokines like TNF, IL-1, and IL-6, which can contribute to persistent joint inflammation. During RA progression, dynamic changes in DNA methylation status are observed in patients’ synovial fibroblasts. Certain cytokines, such as TNF and IL-1β, may downregulate DNMT expression, leading to decreased DNA methylation and enhanced expression of genes associated with RA. This process can result in more aggressive behaviors in FLS, possibly exacerbating RA. Additionally, pro-inflammatory cytokines like TNF, IL-6, and IL-17 can activate synovial fibroblasts to release various pathogenic molecules, including IL-6, GM-CSF, and MMP, which further amplify inflammation. These cytokines also stimulate the production of RANKL in fibroblasts. IL-17-activated immune cells can further increase RANKL expression in synovial fibroblasts. RANKL interacts with its receptor to encourage macrophages to become osteoclasts, potentially leading to bone erosion in RA. In OA inflammatory mediators such as TNF, IL-1, and IL-6 are thought to upregulate MMPs and chondro-degradases, contributing to the breakdown of articular cartilage and reducing the synthesis of collagen and proteoglycan. PGE2, a key mediator of inflammation in OA, is produced by COX. The same inflammatory cytokines activate the p38/NF-kB pathway, which plays a role in inflammation development. In the synovium, IL-1, TNF-α, and other factors may cause overexpression of iNOS and its product, NO, in OA chondrocytes. NO can exacerbate OA by increasing proteoglycan degradation and inhibiting the synthesis of collagen and proteoglycan in chondrocytes. Moreover, noise exposure can lead to an overproduction of ROS, which are thought to be primary factors in noise-induced inflammation. Elevated ROS levels can induce the production of NF-κB and IL-1β, while simultaneously downregulating the intracellular peroxisome PPAR. The reduced activity of PPAR may further enhance the production of inflammatory factors, contributing to the progression of OA. SNS, sympathetic nervous system; PNS, parasympathetic nervous system; ANS, autonomic nervous system; TNF, tumor necrosis factor; IL, interleukin; RA, rheumatoid arthritis; DMNT, DNA methyltransferase; FLS, fibroblast like synoviocytes; MMPs, metalloproteinases; GM-CSF, granulocyte-macrophage colony-stimulating factor; RANKL, receptor activator of nuclear factor-κB ligand; OA, osteoarthritis; PGE2, prostaglandin E2; COX, cyclooxygenase; iNOS, inducible nitric oxide synthase; NO, nitric oxide; ROS, reactive oxygen species; PPAR, proliferator-activated receptor

To our knowledge, this is the first study to simultaneously explore the association between occupational noise exposure and the prevalence of OA and RA among American respondents. A previous study utilizing the NHANES database to investigate the association between occupational noise exposure and the prevalence of RA had results similar to ours [15]. Dzhambov et al. reported an increased risk of RA among individuals exposed to occupational noise for more than 15 years and hypothesized that occupational noise exposure exceeding 85 dB(A) may be a potential risk factor for RA. Our findings provide additional evidence for an association between occupational noise exposure and RA as well as OA. We included all NHANES data reporting the duration of occupational noise exposure and adjusted for additional potential confounders, revealing that individuals exposed to occupational noise for more than 1 year had a significantly increased risk of RA. In addition, we performed subgroup analyses and explored potential mechanisms for the association between noise and arthritis below (Details are shown in Fig. 3). Nevertheless, a prior case‒control study examining community traffic noise failed to establish a significant association between RA and noise exposure, possibly due to the interference of other risk factors that may be more prevalent near roadways [16]. There is currently a lack of direct evidence for the impact of occupational noise exposure on OA. Some evidence suggests a positive relationship between tinnitus and OA, and occupational noise exposure is a proven risk factor for tinnitus [41–43], which may explain this relationship.

Mechanistically, exposure to noise disrupts the balance of the autonomic nervous system, leading to a decrease in the release of acetylcholine, thereby causing an increase in the production of proinflammatory cytokines such as TNF, IL-1 and IL-6 [17–20]. Inflammation may be a common mechanistic pathway involved in RA and OA. During RA progression, cytokines such as TNF and IL-1β downregulate DNA methyltransferase expression, reducing DNA methylation and increasing RA-associated gene expression. This leads to more aggressive fibroblast-like synovial cell behaviours, possibly worsening RA [44, 45]. Additionally, TNF-α, IL-6, and IL-17 activate synovial fibroblasts, enhancing the production of pathogenic molecules such as IL-6, GM-CSF, and MMPs [46–48] and upregulating RANKL expression, ultimately contributing to joint damage [49, 50]. In addition to RA, a range of inflammatory factors might contribute to the development of OA by promoting the degradation of proteoglycans [51, 52] and inhibiting collagen synthesis [53–55]. Furthermore, noise exposure could increase the production of reactive oxygen species, potentially triggering the production of NF-κB and IL-1β and reducing the levels of peroxisome proliferator-activated receptors, possibly further exacerbating inflammation and accelerating the development of OA [56]. The mechanism above is shown in Fig. 3.

Age was identified as a significant risk factor influencing the relationship between occupational noise and OA. The results showed that individuals aged 40 and younger who are exposed to noise had higher odds of self-reported OA compared to those who are older than 40 years. The effect of age on the occurrence of OA may be related to the appropriate age of individuals in noise-exposed occupations [57]. Clinically, the onset of OA at 40 years is considered early [58]. Our study revealed a stronger link between occupational noise exposure and OA in individuals aged younger than 40 years than in those aged 40 years and older. One speculation is that the intensity of work in certain roles contributes to a predisposition to injuries [59], which might be more prevalent in jobs that employ people aged 40 and younger [57].

For the noninstitutionalized population of the United States, the NHANES includes a large sample from diverse ethnic backgrounds, which is an advantage of our study. Only one previous study has specifically addressed the potential association between RA and occupational noise exposure [15], and no reports on OA have been found. In comparison with this study [15], our findings add more evidence for an association between occupation noise exposure and RA and also is the first to report on an association between occupation noise exposure and OA, filling a gap in the related research. However, certain limitations in this study must be acknowledged. First, this study identified the association between occupational noise exposure and OA/RA but could not establish causality due to its cross-sectional nature. Second, the majority of variables were collected from questionnaire responses, which introduced the potential for recall bias and limitations in the accuracy of the results due to the subjective nature of self-reports, warranting caution in the interpretation of the findings. Third, considering the absence of specific information regarding the timing of occupational noise exposure and arthritis diagnosis, it is possible that noise exposure might have occurred subsequent to the diagnosis of RA or OA, potentially affecting our conclusions. Fourth, occupational noise exposure may serve as a proxy for certain unmeasured variables in the workplace that impact the onset of arthritis.

## Conclusion

In conclusion, this study revealed a positive association between occupational noise exposure and the prevalence of RA and OA within a diverse cohort of American individuals aged ≥ 20 years. However, further confirmation through prospective cohort studies is necessary. If substantiated, such an association would carry profound implications for public health.

### Supplementary Information

**Additional file 1: Supplementary Table 1.** The characteristics of respondents with occupational noise exposure duration from the 2011-2012, 2015-March 2020 Pre-pandemic NHANES.

**Additional file 2: Supplementary Table 2.** Variance Inflation Factor (VIF) test for each confounding factors.

**Additional file 3: Supplementary Table 3.** Prevalence of OA and RA in occupational noise-exposed and control populations at different ages.

**Additional file 4: Supplementary Table 4.** Prevalence of OA and RA in respondents with different occupational noise exposure duration.

**Additional file 5: Supplementary Table 5.** ORs with confidence intervals for the confounding factors in the fully adjusted models of Multivariate logistic regression.

## Data Availability

The NHANES repository contains the datasets generated and analyzed for the current study. This data can be accessed via the link below and is available for download: https://wwwn.cdc.gov/nchs/nhanes/Default.aspx.

## Abbreviations

OA: Osteoarthritis
RA: Rheumatoid arthritis
NHANES: National Health and Nutrition Examination Survey
BMI: Body mass index
MET: Metabolic equivalent
IL-17: Interleukin-17
