# Supplementary material for: Is occupational noise associated with arthritis? Cross-sectional evidence from US population
Source: BMC Public Health. 2024 Feb 5;24:371. doi: 10.1186/s12889-024-17897-0 (PMC10840213; doi:10.1186/s12889-024-17897-0)
Supplement: Supplementary file 1 — Additional file 1: Supplementary Table 1. The characteristics of respondents with occupational noise exposure duration from the 2011-2012, 2015-March 2020 Pre-pandemic NHANES. [file 12889_2024_17897_MOESM1_ESM.docx]

**Supplementary Table 1**. The characteristics of respondents with occupational noise exposure duration from the 2011-2012, 2015-March 2020 Pre-pandemic NHANES

|  | **Control Group**  **(n=** **6515)** | **Occupational Noise Exposure (n=3168))** | ***P* Value** |
| --- | --- | --- | --- |
| Osteoarthrosis |  |  | 0.034 |
| Yes | 8.66% | 9.97% |  |
| No | 91.34% | 90.03% |  |
| Rheumatoid arthritis |  |  | <0.001* |
| Yes | 3.73% | 6.09% |  |
| No | 96.27% | 93.91% |  |
| Age (year) | 46.49 ± 17.03 | 48.42 ± 16.90 | <0.001* |
| Gender |  |  | <0.001* |
| Male | 38.63% | 69.26% |  |
| Female | 61.37% | 30.74% |  |
| Race |  |  | <0.001* |
| Latino | 25.73% | 28.54% |  |
| Non-Hispanic white | 30.73% | 39.46% |  |
| African American | 24.82% | 26.07% |  |
| Asian | 18.73% | 5.93% |  |
| Education level |  |  | <0.001* |
| Less than high school | 20.29% | 27.78% |  |
| High school or above | 79.71% | 72.22% |  |
| Marital status |  |  | 0.237 |
| Married/Living with Partner | 58.53% | 59.79% |  |
| Unmarried | 41.47% | 40.21% |  |
| Body mass index (kg/m^2^) | 28.86 ± 6.88 | 29.78 ± 6.55 | <0.001* |
| Ratio of family income to poverty | 2.54 ± 1.59 | 2.21 ± 1.44 | <0.001* |
| Hypertension |  |  | 0.030* |
| Yes | 8.96% | 10.29% |  |
| No | 39.28% | 40.34% |  |
| Missing | 51.76% | 49.37% |  |
| Diabetes |  |  | <0.001* |
| Yes | 11.30% | 15.88% |  |
| No | 88.70% | 84.12% |  |
| Thyroid problem |  |  | 0.003* |
| Yes | 10.01% | 8.11% |  |
| No | 89.99% | 91.89% |  |
| Sleep trouble |  |  | <0.001* |
| Yes | 22.50% | 27.05% |  |
| No | 77.50% | 72.95% |  |
| Smoke |  |  | <0.001* |
| Yes | 34.12% | 53.44% |  |
| No | 65.88% | 46.56% |  |
| Alcohol |  |  | <0.001* |
| Yes | 52.86% | 65.56% |  |
| No | 28.40% | 20.99% |  |
| Missing | 18.74% | 13.45% |  |
| METs | 2637.79 ± 4566.25 | 5462.43 ± 7805.95 | <0.001* |

*: Significant at the *P* < 0.05 level

Mean±SD for continuous variables: *P* value was calculated by the t-test; % for categorical variables, *P* value was calculated by the chi-square test. MET, metabolic equivalent.
