# Supplementary material for: Is occupational noise associated with arthritis? Cross-sectional evidence from US population
Source: BMC Public Health. 2024 Feb 5;24:371. doi: 10.1186/s12889-024-17897-0 (PMC10840213; doi:10.1186/s12889-024-17897-0)
Supplement: Supplementary file 2 — Additional file 2: Supplementary Table 2. Variance Inflation Factor (VIF) test for each confounding factors. [file 12889_2024_17897_MOESM2_ESM.docx]

**Supplementary Table 2.** Variance Inflation Factor (VIF) test for each confounding factors

| Confounding factors | VIF |
| --- | --- |
| Race | 1.106 |
| Gender | 1.162 |
| Age | 1.201 |
| Education | 1.164 |
| Marriage | 1.057 |
| Income | 1.176 |
| Hypertension | 1.044 |
| BMI | 1.083 |
| Diabetes | 1.124 |
| Thyroid disease | 1.085 |
| METs | 1.079 |
| Sleep trouble | 1.055 |
| Smoke | 1.121 |
| Alcohol | 1.046 |

The variance expansion coefficient (VIF) is less than 5, indicating that there is no collinearity relationship between confounding factors.
