# Supplementary material for: Is occupational noise associated with arthritis? Cross-sectional evidence from US population
Source: BMC Public Health. 2024 Feb 5;24:371. doi: 10.1186/s12889-024-17897-0 (PMC10840213; doi:10.1186/s12889-024-17897-0)
Supplement: Supplementary file 3 — Additional file 3: Supplementary Table 3. Prevalence of OA and RA in occupational noise-exposed and control populations at different ages. [file 12889_2024_17897_MOESM3_ESM.docx]

**Supplementary Table 3.** Prevalence of OA and RA in occupational noise-exposed and control populations at different ages.

| ***Age stratification*** | Self-Reported RA | | | Self-Reported OA | | |
| --- | --- | --- | --- | --- | --- | --- |
|  | Control group | Occupational noise | *P*-value | Control group | Occupational noise | *P*-value |
| <40 | 0.78% | 1.25% | 0.17 | 1.01% | 3.21% | <0.01 |
| ≥40, <60 | 3.99% | 6.19% | <0.01 | 7.54% | 10.01% | 0.02 |
| ≥60 | 8.20% | 11.23% | <0.01 | 20.62% | 17.48% | 0.02 |

*P* value was calculated by chi-square test.
