# Supplementary material for: Is occupational noise associated with arthritis? Cross-sectional evidence from US population
Source: BMC Public Health. 2024 Feb 5;24:371. doi: 10.1186/s12889-024-17897-0 (PMC10840213; doi:10.1186/s12889-024-17897-0)
Supplement: Supplementary file 4 — Additional file 4: Supplementary Table 4. Prevalence of OA and RA in respondents with different occupational noise exposure duration. [file 12889_2024_17897_MOESM4_ESM.docx]

**Supplementary Table 4.** Prevalence of OA and RA in respondents with different occupational noise exposure duration.

| *Occupational noise exposure duration* | Self-Reported RA | | Self-Reported OA | |
| --- | --- | --- | --- | --- |
|  | N | % | N | % |
| No history of exposure | 564 | 8.7% | 243 | 3.7% |
| Less than 1 year | 38 | 6.9% | 16 | 2.9% |
| 1 to 4 years | 78 | 8.8% | 51 | 5.7% |
| 5 to 14 years | 86 | 9.6% | 53 | 5.9% |
| 15 or more years | 114 | 13.7% | 73 | 8.8% |

N indicates the number of persons with the disease at that exposure duration stage, and % indicates the prevalence rate at that stage.
