# Supplementary material for: Is occupational noise associated with arthritis? Cross-sectional evidence from US population
Source: BMC Public Health. 2024 Feb 5;24:371. doi: 10.1186/s12889-024-17897-0 (PMC10840213; doi:10.1186/s12889-024-17897-0)
Supplement: Supplementary file 5 — Additional file 5: Supplementary Table 5. ORs with confidence intervals for the confounding factors in the fully adjusted models of Multivariate logistic regression. [file 12889_2024_17897_MOESM5_ESM.docx]

**Supplementary Table 5.** ORs with confidence intervals for the confounding factors in the fully adjusted models of Multivariate logistic regression.

|  | Self-Reported RA | | Self-Reported OA | |
| --- | --- | --- | --- | --- |
|  | OR (95% CI) | *P* value | OR (95% CI) | *P* value |
| Race |  |  |  |  |
| Latino | reference |  | reference |  |
| Non-Hispanic white | 0.68 (0.53, 0.86) | <0.01 | 1.67 (1.38, 2.02) | <0.01* |
| African American | 1.29 (1.01, 1.64) | 0.04 | 1.08 (0.87, 1.34) | 0.51 |
| Asian | 0.55 (0.35, 0.86) | <0.01 | 1.05 (0.76, 1.46) | 0.76 |
| Gender |  |  |  |  |
| Male | reference |  | reference |  |
| Female | 1.42 (1.15, 1.75) | <0.01 | 2.35 (2.00, 2.77) | <0.01* |
| Age | 1.05 (1.04, 1.05) | <0.01 | 1.06 (1.05, 1.06) | <0.01* |
| Education level |  |  |  |  |
| Less than high school | reference |  | reference |  |
| High school or above | 0.79 (0.64, 0.97) | 0.02 | 1.27 (1.06, 1.52) | 0.01* |
| Marital status |  |  |  |  |
| Married/Living with Partner | reference |  | reference |  |
| Unmarried | 1.24(1.03, 1.50) | 0.02 | 1.13(0.98, 1.30) | 0.10 |
| Ratio of family income to poverty | 0.90(0.84, 0.96) | <0.01 | 1.02(0.97, 1.07) | 0.49 |
| Hypertension |  |  |  |  |
| No | reference |  | reference |  |
| Yes | 0.83 (0.65, 1.07) | 0.15 | 0.87 (0.72, 1.05) | 0.14 |
| Missing | 0.98 (0.80, 1.20) | 0.84 | 1.07 (0.92, 1.26) | 0.39 |
| BMI | 1.02 (1.01, 1.03) | <0.01 | 1.04 (1.03, 1.05) | <0.01* |
| Diabetes |  |  |  |  |
| No | reference |  | reference |  |
| Yes | 1.38(1.12, 1.70) | <0.01 | 1.12(0.95, 1.33) | 0.19 |
| Thyroid problem |  |  |  |  |
| No | reference |  | reference |  |
| Yes | 1.17 (0.92, 1.49) | 0.20 | 1.45 (1.22, 1.73) | <0.01* |
| METs | 1.00 (1.00, 1.00) | 0.01 | 1.00 (1.00, 1.00) | 0.89 |
| Sleep trouble |  |  |  |  |
| No | reference |  | reference |  |
| Yes | 1.90 (1.58, 2.29) | <0.01 | 2.20 (1.91, 2.52) | <0.01* |
| Smoke |  |  |  |  |
| No | reference |  | reference |  |
| Yes | 1.14 (0.95, 1.38) | 0.17 | 1.26 (1.09, 1.46) | <0.01* |
| Alcohol |  |  |  |  |
| No | reference |  | reference |  |
| Yes | 0.93 (0.75, 1.14) | 0.48 | 1.24 (1.05, 1.46) | 0.01* |
| Missing | 0.75 (0.57, 0.99) | 0.04 | 0.90 (0.72, 1.11) | 0.32 |

*: Significant at the *P* < 0.05 level

The data: OR (95% CI), *P* value. Fully adjusted model: age, BMI, alcohol, gender, race, education level, marital status, hypertension, diabetes, ratio of family income to poverty, sleep trouble, smoke, METs, thyroid disease were adjusted; OR, odds ratio; MET, metabolic equivalent; BMI, body mass index.
